# Prognostic Impact of Main Pulmonary Artery Enlargement in Patients Undergoing Transcatheter Aortic Valve Replacement

**DOI:** 10.1016/j.cjco.2026.05.008

**Published:** 2026-05-26

**Authors:** Dionysios Adamopoulos, Quentin Liabot, Léon Genecand, Christina Gkotsoulia, Sarah Mauler-Wittwer, Georgios Giannakopoulos, Jean-François Deux, Jakub Walenczak, Georgios Rovas, Marc Arcens, Lindsey Crowe, Hajo Müller, Nikolaos Stergiopulos, Jean-Paul Vallée, Stéphane Noble

**Affiliations:** a Faculty of Medicine, Department of Medicine, Geneva University, Geneva, Switzerland; b Department of Internal Medicine, Division of Cardiology, Hôpitaux Universitaires de Genève (HUG), Geneva, Switzerland; c Department of Diagnostics, Division of Nuclear Medicine, Hôpitaux Universitaires de Genève (HUG), Geneva, Switzerland; d Department of Internal Medicine, Division of Pneumology, Hôpitaux Universitaires de Genève (HUG), Geneva, Switzerland; e Pulmonary Hypertension Program, Hôpitaux Universitaires de Genève (HUG), Geneva, Switzerland; f Department of Diagnostics, Division of Radiology, Hôpitaux Universitaires de Genève (HUG), Geneva, Switzerland; g Laboratory of Hemodynamics and Cardiovascular Technology, Ecole Polytechnique Fédérale de Lausanne (EPFL), Lausanne, Switzerland

**Keywords:** transcatheter aortic valve implantation, transcatheter aortic valve replacement, pulmonary hypertension, aortic valve stenosis, main pulmonary artery enlargement

## Abstract

**Background:**

Pulmonary hypertension is characterized by extensive remodelling of the pulmonary tree, with enlargement of the pulmonary arteries. The predictive value of pulmonary hypertension in patients undergoing transcatheter aortic valve replacement (TAVR) has been demonstrated. We aimed to evaluate the prognostic impact of the pre-procedural main pulmonary artery (mPA) enlargement, assessed by computed tomography angiography, in patients undergoing TAVR for severe aortic stenosis (AS).

**Methods:**

We retrospectively analyzed 337 consecutive patients with AS from our local registry who underwent right heart catheterization and computed tomography angiography before TAVR. The cohort was divided into 2 groups according to the presence of an enlarged mPA (mPA axial diameter ≥ 29 mm for male patients and ≥ 27 mm for female patients, mPA_e_, n = 192, mean age 83 ± 6 years, 41% male) or not (mPA_n_, n = 145, mean age 84 ± 6 years, 46% male). The primary endpoint was all-cause mortality at 1 year.

**Results:**

A significant correlation between invasive mean pulmonary artery pressure and mPA axial diameter was noted (Pearson *r* = 0.393, *P* < 0.001). Compared with the mPA_n_ group, patients with an enlarged mPA had higher 1-year mortality rates (hazard ratio [HR]: 2.59, 95% confidence interval [CI]: 1.11-6.04, *P* = 0.027). The prognostic significance was even stronger after indexing the mPA diameter to the ascending aortic diameter (HR 3.83, 95% CI: 1.85-7.87, *P* < 0.001). This measure remained significant after adjustment for baseline comorbidities (adjusted HR 4.2; 95% CI: 2.00-8.98, *P* < 0.001).

**Conclusions:**

Pre-procedural mPA enlargement is strongly associated with 1-year all-cause mortality in patients undergoing TAVR. This finding may pave the way for a more precise noninvasive risk stratification in patients with severe AS.

## Central Illustration

**Figure:**
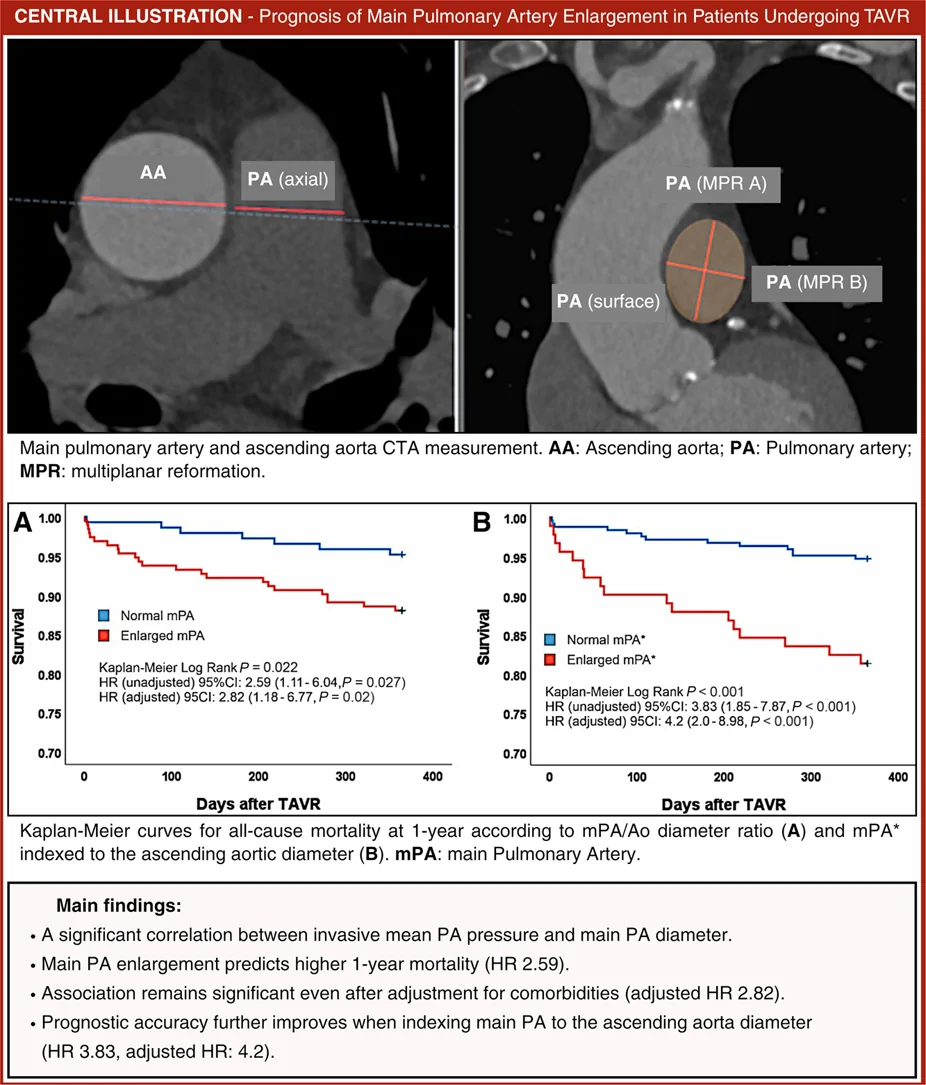

## Résumé

**Contexte:**

L'hypertension pulmonaire (HP) se caractérise par un remodelage important de l'arbre pulmonaire, accompagné d'une dilatation des artères pulmonaires (AP). La valeur pronostique de l'HP chez les patients bénéficiant d'un remplacement valvulaire aortique par cathéter (RVAC) a déjà été démontrée. Notre objectif était d'évaluer l'impact pronostique d'une dilatation préprocédurale de l'artère pulmonaire principale (APP), évaluée par angiographie par tomodensitométrie (angio-TDM), chez les patients bénéficiant d'un RVAT pour une sténose aortique (SA) sévère.

**Méthodologie:**

Nous avons analysé rétrospectivement 337 patients consécutifs atteints de SA, issus de notre registre local, et ayant bénéficié d’un cathétérisme cardiaque droit et une angio-TDM avant le RVAC. La cohorte a été divisée en deux groupes selon la présence d’une APP dilatée (diamètre axial de l’APP ≥ 29 mm chez les hommes et ≥ 27 mm chez les femmes, APPd, n = 192, âge moyen 83 ± 6 ans, 41 % d’hommes) ou non (APPnd, n = 145, âge moyen 84 ± 6 ans, 46 % d’hommes). Le critère d'évaluation principal était la mortalité toutes causes confondues à 1 an.

**Résultats:**

Une corrélation significative a été observée entre la pression artérielle pulmonaire moyenne invasive et le diamètre axial de l’APP (coefficient *r* de Pearson = 0,393, *p* < 0,001). Par rapport au groupe APPnd, les patients présentant une APP dilatée avaient des taux de mortalité à 1 an plus élevés (rapport des risques instantanés [RRi] : 2,59, intervalle de confiance [IC] à 95 % : 1,11-6,04, *p* = 0,027). La signification pronostique était encore plus forte après indexation du diamètre de l’artère pulmonaire sur le diamètre de l’aorte ascendante (RRi 3,83, IC à 95 % : 1,85-7,87, *p* < 0,001). Cette association demeurait significative après ajustement pour les comorbidités de base (RRi ajustés 4,2; IC à 95 % : 2,00-8,98, *p* < 0,001).

**Conclusions:**

Une dilatation préprocédurale de l’APP est fortement associée à la mortalité toutes causes confondues à 1 an chez les patients bénéficiant d’un RVAC. Cela pourrait ouvrir la voie à une stratification non invasive plus précise du risque chez les patients atteints d’une SA sévère.

Pulmonary hypertension (PH) has been recognized as one of the major predictors of mortality in patients undergoing transcatheter aortic valve replacement (TAVR) due to severe aortic stenosis (AS). Worse outcomes following TAVR, such as increased mortality or higher postprocedural complication rates, are observed in patients with elevated pulmonary artery pressure (PAP), especially those with a significant pre-capillary component due to increased pulmonary vascular resistance.^1^, ^2^, ^3^, ^4^, ^5^, ^6^, ^7^ Our group demonstrated that the presence of PH (mean PAP pressure ≥ 20 mm Hg) was associated with higher 1-year mortality rates, compared to those in patients without PH, even after adjusting for baseline **Euro**pean **S**ystem for **C**ardiac **O**perative **R**isk **E**valuation (EuroSCORE) values.^2^ In accordance, O’Sullivan et al. showed that PH is an important predictor of 1-year mortality after TAVR, with a hazard ratio of approximately 2, even after adjusting for baseline comorbidities.^1^

Right heart catheterization (RHC) is the gold standard for diagnosing PH, as it provides highly accurate and comprehensive hemodynamic measurements in patients with severe AS. Despite its invasive nature, the risk of RHC complications, namely arrhythmias and bleeding, which can be reduced by use of a brachial approach or systematic ultrasound guidance for femoral puncture, remains low. However, these complications may be of concern in frail patients with severe AS.^8^ In addition, the RHC is performed at the time of the coronary angiogram, which is increasingly being performed only at the time of TAVR.^9^ The latest guidelines of the European Society of Cardiology on valvular heart disease even recommend, as a class IIa, the omission of pre-TAVR invasive coronary angiography if the pre-TAVR computed tomography angiography (CTA) is of sufficient quality to rule out significant coronary artery disease.^10^ In this context, interest is emerging in the use of noninvasive techniques such as CTA to assess the presence of PH. This interest is particularly relevant as CTA-derived main pulmonary artery (mPA) enlargement correlates accurately with the presence of PH.^11^

Very few studies have assessed the prognostic significance of CTA-derived mPA enlargement in patients with severe AS.^12^^,^^13^ We therefore aimed to assess the effect of baseline, pre-procedural CTA-derived mPA diameter on clinical outcomes in patients with AS treated with TAVR.

## Materials and Methods

### Study population

We retrospectively analyzed medical records of patients who underwent TAVR at our institution between June 2008 and December 2019. The study population comprised patients with symptomatic native-valve AS at high or intermediate risk for conventional surgical aortic valve replacement.

**Figure 1:**
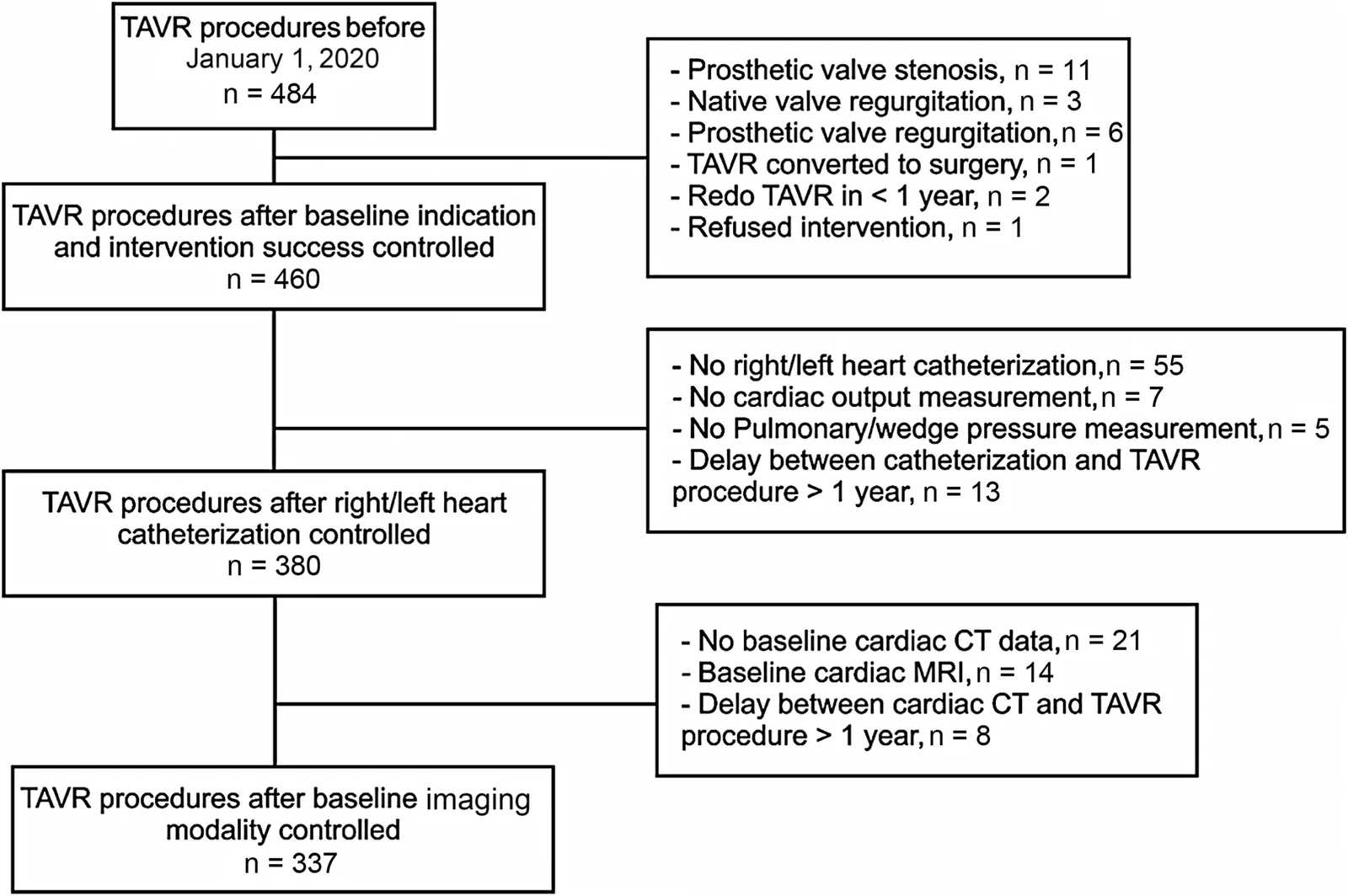
Flow chart of the study population. CT, computed tomography; MRI, magnetic resonance imaging; TAVR, transcatheter aortic valve replacement.

Of 484 patients, 380 (78%) had a baseline RHC and a successful TAVR and were included in the study. An additional 43 patients were excluded because no baseline CTA was available or because of a significant delay between the CTA and the TAVR procedure (delay > 1 year; Fig. 1). The final cohort consisted of 337 patients, divided into 2 groups based on mPA diameter. We defined mPA enlargement as exceeding 29 mm in male patients and 27 mm in female patients.^10^ Data were anonymized before the analysis. All patients signed a written informed consent form for our local registry, part of the Swiss prospective registry (approved by the local ethics committee, NCT01368250), and for the use of their coded data for research purposes in accordance with the ethical principles of the Helsinki Declaration and applicable regulations. A detailed study flowchart is presented in Figure 1.

### Baseline echocardiographic assessment

Transthoracic echocardiography was performed in all study participants before the procedure. We allocated the acquired images to a dedicated workstation for further offline analysis (IntelliSpace Cardiovascular 5.1, Philips Medical Systems Nederland BV, Best, Netherlands).

The study recorded left ventricular (LV) geometry (LV end-diastolic diameter, LV mass indexed to body surface area [BSA]) and calculated LV mass using the Devereux formula. Ejection fraction (EF) was visually estimated. For the diastolic function, we estimated the mitral E wave peak velocity (n = 333), mitral A wave peak velocity (n = 262), e' mean (n = 330), and left atrial volume indexed to BSA (n = 375). Right ventricular (RV) function was assessed by tricuspid annular plane systolic excursion and pulsed Doppler peak velocity at the tricuspid annulus (TDI). Tricuspid regurgitation was systematically assessed in all patients using color Doppler. For the AS evaluation, we estimated the transvalvular mean and maximum pressure gradients. The aortic valve area was calculated using the continuity equation and indexed to BSA. The staging of the AS was performed using the standard criteria established by Genereux et al.^14^ Evaluation of all valves was performed using the standard echocardiographic views and according to the established criteria proposed by the European Society of Cardiology.^15^

### Invasive hemodynamics

As part of the standard evaluation, each patient had a pre-procedural RHC. We used the Seldinger technique to place a 7F Swan-Ganz or a 6F Arrow balloon-tip catheter in the brachial or femoral vein. Cardiac output (CO) for each patient was measured by either the indirect Fick method and/or the thermodilution technique. Thereafter, CO was adjusted for BSA to derive the cardiac index (CI). Stroke volume (SV) was derived by dividing CO by heart rate and adjusting for BSA to calculate the stroke volume index. The zero-reference level was placed at the mid-thoracic line, corresponding to the level of the posterior wall of the right atrium. Standard measurements included the systolic, diastolic, mean pulmonary artery pressure, pulmonary artery wedge pressure (PAWP), transpulmonary pressure gradient , pulmonary arterial compliance, and pulmonary vascular resistance. All pressure measurements were performed using fluid-recording catheters connected to pressure transducers.

### CTA assessment

All patients underwent a CTA scan to assess the size of the aortic annulus and vascular access using a dual-source CT scanner (Somatom Drive, Siemens Healthcare, Erlangen, Germany). A specialized radiologist in cardiovascular imaging from our radiology department (J.W.) analyzed the images and performed PA measurements, blinded to the RHC results. The mPA diameter is conventionally measured at its bifurcation, perpendicular to its long axis, on an axial slice (Central Illustration). An mPA diameter > 29 mm for male patients and > 27 mm for female patients has been used traditionally as a threshold above which PH is suggested, with a 97% positive predictive value, 89% specificity, and 87% sensitivity.^10^ Values of the mPA also have been normalized to the ascending aortic diameter measured at the level of the mPA, with a cutoff value of 0.91 defining PA enlargement (Central Illustration). Two additional diameters of the PA, as well as the PA surface, were additionally measured after multiplanar reformation.

**Central Illustration:**
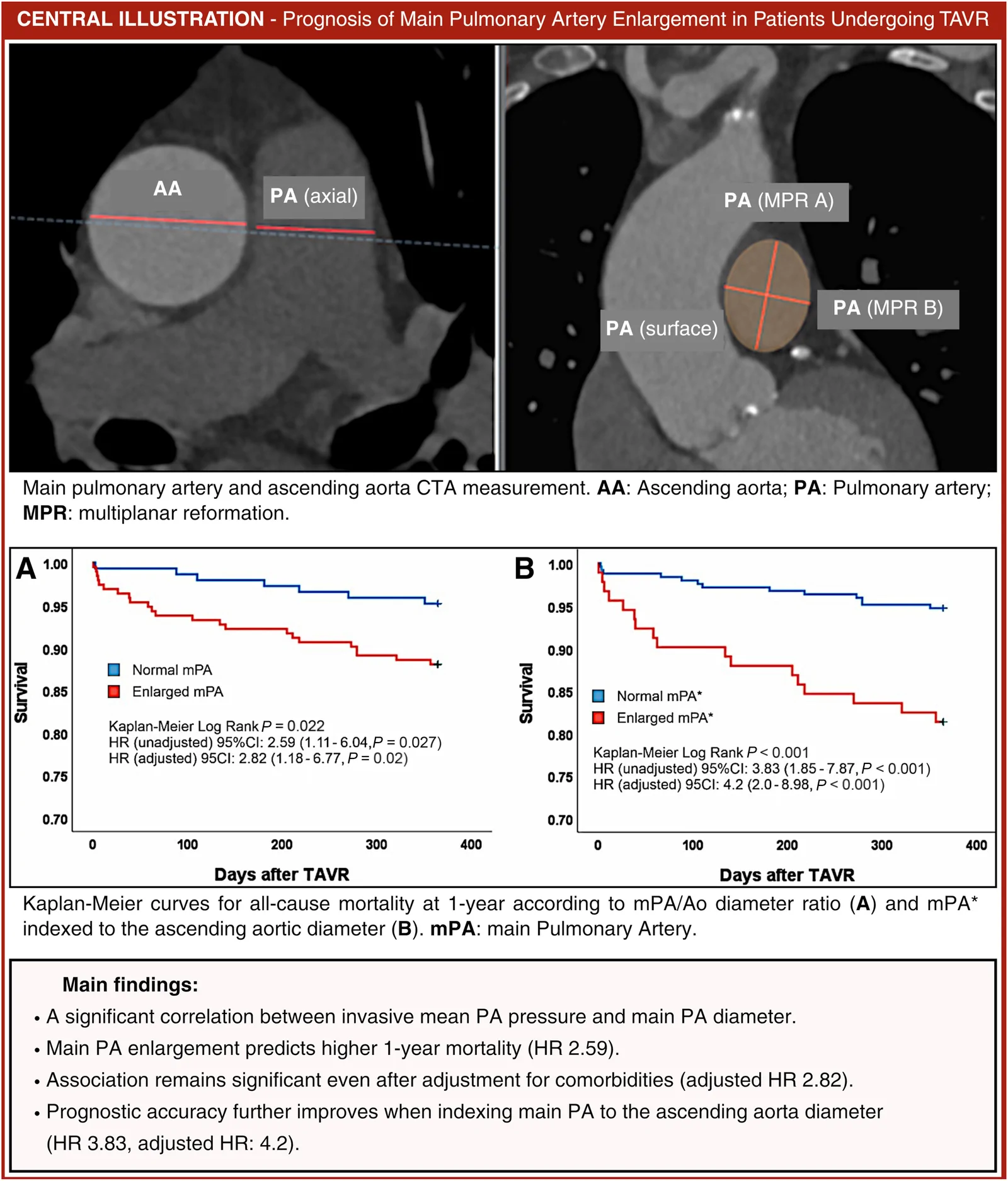
Prognostic impact of main pulmonary artery (mPA) enlargement in patients undergoing transcatheter aortic valve replacement (TAVR). AA, ascending aorta; Ao, aorta; CI, confidence interval; CTA, computed tomography angiography; HR, hazard ratio; mPAP, mean pulmonary artery pressue; MPR, multiplanar reformation; PA, pulmonary artery.

### Procedure characteristics

Aortic valve replacement was performed by implantation of either the self-expanding CoreValve/Evolut (Medtronic, Minneapolis, MN, n = 302; 90%), the Edwards Sapien S3 (Edwards Lifesciences, Irvine, CA n = 30; 8.5%), or the ACURATE neo (Boston Scientific, Marlborough, MA, n = 5; 1.5%). Device implantation success was systematically evaluated for all interventions according to the Valve Academic Research Consortium-2 consensus document criteria.^16^

### Follow-up

All patients were followed up in the post-TAVR period at 1, 6, and 12 months by clinical visits performed by their external cardiologists (1 and 12 months) and at our outpatient clinic (at 6 months). All data were entered into an encrypted online database (www.openclinica.com, OpenClinica, Waltham, MA). The primary endpoint for the study was 1-year all-cause mortality. All events were adjudicated by an independent clinical committee.

### Statistical analysis

The study population was divided into 2 groups according to the mPA diameter: (i) patients with normal mPA [mPA_n_], n = 145; 43%; and (ii) enlarged mPA [mPA_e_], n = 192; 57%. An additional categorization of the study population was performed according to the mPA diameter normalized to the ascending aorta size (normal mPA∗, n = 246; 73%; enlarged mPA, n = 91; 27%). Categorical variables are expressed as counts with percentages. Continuous variables are expressed as mean values ± standard deviation or as median and interquartile range in case of violation of the normal distribution (assessed by visual inspection of histogram frequencies). Categorical variables are compared between the 2 groups using the Pearson χ^2^ or Fisher's exact test, as appropriate. For continuous variables, comparisons among groups were performed using a simple *t* test. The associations among mPA diameter, PAP, and RV performance indices were assessed using Pearson correlation coefficients. One-year all-cause mortality rates for the 2 groups were calculated from Kaplan–Meier analysis (Central Illustration). Cox regression analysis was performed to compute HRs and 95% CIs. A multivariate Cox regression model (enter method) was used to adjust comparisons between groups. The model included the following parameters: age, diabetes, chronic obstructive pulmonary disease (COPD), body mass index (BMI), and atrial fibrillation. In addition, an extended Cox regression model was constructed, including established predictors of mortality in TAVR populations, namely age, sex, coronary artery disease, COPD, renal failure, and procedural access. Statistical significance was assumed at a 2-sided *P*-value level of 0.05. Statistical analysis was performed in IBM SPSS, version 27.0 (IBM, Armonk, NY).

## Results

### Baseline characteristics

The baseline characteristics of the study population by mPA group are presented in Table 1. Patients in the mPA_e_ group were younger (aged 83 ± 6 vs 84 ± 6 years, *P* = 0.017) and had higher weight (74 ± 16 vs 68 ± 13 kg, *P* < 0.001) and BMI (27 ± 5.5 vs 25 ± 3.9 kg/m^2^, *P* < 0.001) than those in the mPA_n_ group. Atrial fibrillation and flutter (39% vs 22%, *P* = 0.001) were more prevalent in the mPA_e_ group, likely driving higher rates of use of oral anticoagulation (35% vs 23%, *P* = 0.018) and calcium-channel blockers (24% vs 15%, *P* = 0.047). Syncope was observed more frequently in the mPA_n_ group (18% vs 8%, *P* = 0.008), whereas other symptoms were comparable. Overall procedural risk scores (EuroSCORE and Society of Thoracic Surgeons [STS] score) were higher in the mPA_e_ group (Table 1).

**Table 1:** Baseline characteristics of the study population by main pulmonary artery (mPA) group

Characteristics	Normal mPA	Enlarged mPA	*P*
	*n = 145*	*n = 192*	
**Demographics**			
Age (Y)	84 ± 6	83 ± 6	0.017
Height, cm	164 ± 9	165 ± 8	0.926
Weight, kg	68 ± 13	74 ± 16	< 0.001
BMI, kg/m^2^	25 ± 3.9	27 ± 5.5	< 0.001
BSA, m^2^	1.75 ± 0.19	1.82 ± 0.22	0.003
Gender, male)	67 (46)	78 (41)	0.306
EuroSCORE, % (n = 330)	12.8 [8.4–18.0]	14.2 [9.6–42.2]	0.022
STS Score, % (n = 329)	4.1 [2.9–8.9]	5.3 [3.5–11.3]	< 0.001
Diabetes	32 (22)	65 (34)	0.018
Dyslipidemia	98 (68)	135 (70)	0.592
Arterial hypertension	108 (75)	161 (84)	0.034
Smokers	10 (7)	17 (9)	0.512
CAD	81 (56)	100 (52)	0.491
Previous MI	16 (11)	26 (14)	0.490
PAD	17 (12)	26 (14)	0.621
COPD	15 (10)	43 (22)	0.004
Renal failure	101 (53)	68 (47)	0.299
Cancer	52 (22)	20 (21)	0.867
Atrial fibrillation/flutter	32 (22)	75 (39)	0.001
**Presence of symptoms**			
NYHA III or IV	99 (68)	146 (76)	0.113
Syncope (n = 331)	25 (18)	15 (8)	0.008
Angina (n = 329)	35 (25)	35 (19)	0.173
**Baseline medications**			
Aspirin	84 (58)	100 (52)	0.286
Oral anticoagulation	34 (23)	68 (35)	0.018
Beta-blockers	49 (34)	85 (44)	0.052
ACE inhibitors	34 (23)	45 (23)	0.998
ARBs	50 (35)	68 (35)	0.859
Calcium-channel blockers	22 (15)	46 (24)	0.047
Statin	80 (55)	110 (57)	0.698

Values are mean (± standard deviation), n (%), or median (interquartile range), unless otherwise indicated.

ACE, angiotensin-converting enzyme; ARB, angiotensin receptor blockers; BMI, body mass index; BSA, body surface area; CAD, coronary artery disease; COPD, chronic obstructive pulmonary disease; EuroSCORE, **Euro**pean **S**ystem for **C**ardiac **O**perative **R**isk **E**valuation ; MI, myocardial infarction; mPA, main pulmonary artery; NYHA, New York Heart Association; PAD, peripheral artery disease; STS, Society of Thoracic Surgeons.

### Echocardiographic and RHC parameters

The 2 groups exhibited equivalent degrees of AS severity, as assessed by transvalvular mean pressure gradient and aortic valve area, measured by both echocardiography and RHC (Tables 2 and 3). AS staging distribution was similar in both groups (*P* = 0.406; Table 3). Patients in the mPA_e_ group presented with more dilated left cardiac chambers and higher LV mass (Table 2). Left atrial volumes were increased in the mPA_e_ group, even after adjusting for BSA (*P* = 0.003). LVEF was comparable between groups (*P* = 0.08), although TDI was lower in the mPA_e_ group (*P* = 0.007). Patients in the mPA_e_ group presented with more severe mitral (*P* = 0.036) and tricuspid regurgitation (*P* = 0.038; Table 2).

**Table 2:** Echocardiographic parameters by main pulmonary artery (mPA) group

Parameters	Normal mPA	Enlarged mPA	*P*
	n = 145	n = 192	
**Aortic valve stenosis severity**			
Transvalvular mean pressure gradient, mm Hg	42 ± 14	41 ± 14	0.700
Transvalvular max pressure gradient, mm Hg	70 ± 22	70 ± 22	0.696
Transvalvular max velocity, cm/s	415 ± 67	412 ± 66	0.727
AVA, cm^2^	0.74 ± 0.21	0.73 ± 0.21	0.906
AVA indexed for BSA, cm^2^/m^2^	0.42 ± 0.12	0.41 ± 0.12	0.242
**LV geometry**			
LV end-diastolic diameter, cm (n = 335)	4.4 ± 0.4	4.8 ± 0.8	< 0.001
LV mass, g (n = 333)	187 ± 62	218 ± 68	< 0.001
LV mass indexed for BSA, g/m^2^ (n = 333)	107 ± 34	120 ± 36	0.001
Relative wall thickness (n = 332)	0.49 ± 0.13	0.45 ± 0.13	0.005
**LV systolic function**			
Ejection fraction, % (n = 336)	63 [39–65]	60 [30–65]	0.080
LVOT flow max, mL/s (n = 351)	243 ± 84	236 ± 78	0.474
**LV diastolic function**			
Mitral E wave maximal velocity, cm/s (n = 333)	85 ± 32	105 ± 36	< 0.001
Mitral A wave maximal velocity, cm/s (n = 262)	107 ± 32	100 ± 39	0.120
e’ mean, m/s (n = 330)	5.4 ± 1.7	5.5 ± 1.9	0.441
Left atrial volume, mL (n = 335)	75 [15–249]	82 [42–238]	0.017
Left atrial volume indexed BSA, mL/m^2^ (n = 375)	41 [7–166]	44 [22–161]	0.003
**RV longitudinal function**			
TAPSE, mm (n = 333)	20 ± 4.3	19 ± 4.9	0.104
TDI, cm/s (n = 330)	11.8 ± 2.9	10.9 ± 2.7	0.007
**Aortic regurgitation**			0.802
None	37 (26)	48 (25)	
Mild	96 (66)	123 (64)	
Mild to moderate	7 (5)	10 (5)	
Moderate	5 (3)	11 (6)	
**Mitral regurgitation**			0.036
None	58 (40)	77 (40)	
Mild	73 (50)	82 (43)	
Mild to moderate	12 (8)	17 (9)	
Moderate	2 (2)	16 (8)	
**Tricuspid regurgitation**			0.038
None	85 (59)	119 (62)	
Mild	50 (35)	45 (23)	
Mild to moderate	7 (5)	13 (7)	
Moderate	3 (2)	10 (5)	
Moderate to severe	0 (0)	5 (3)	

Values are mean (± standard deviation), n (%), or median (interquartile range), unless otherwise indicated.

AVA, aortic valve area; BSA, body surface area; LV, left ventricle; LVOT, left ventricular outflow tract; RV, right ventricle; TAPSE, tricuspid annular plane systolic excursion; TDI, pulsed-wave Doppler peak velocity at the tricuspid annulus;

**Table 3:** Right and left heart catheterization parameters by main pulmonary artery (mPA) group

Parameters	Normal mPA	Enlarged mPA	*P*
	n = 145	n = 192	
**Aortic valve stenosis severity**			
Transvalvular mean pressure gradient, mm Hg (n = 310)	33 ± 14	32 ± 14	0.741
Transvalvular peak to peak pressure gradient, mm Hg (n = 325)	44 ± 20	43 ± 21	0.610
Transvalvular pressure gradient AUC, mm Hgs (n = 310)	14 ± 7	13 ± 6	0.437
AVA, Gorlin, cm^2^ (n = 308)	0.58 ± 0.22	0.57 ± 0.21	0.795
AVA indexed for BSA, Gorlin, cm^2^/m^2^ (n = 308)	0.33 ± 0.11	0.31 ± 0.11	0.381
**Systemic afterload**			
Aortic systolic blood pressure, mm Hg	129 ± 26	121 ± 26	0.009
Aortic diastolic blood pressure, mm Hg	54 ± 11	53 ± 13	0.290
Aortic pulse pressure, mm Hg	74 ± 21	68 ± 22	0.013
Aortic mean pressure, mm Hg	83 ± 16	79 ± 16	0.022
Total arterial compliance, mL/mm Hg (n = 351)	0.55 ± 0.27	0.57 ± 0.27	0.598
Total vascular resistance, mm Hg·min/mL (n = 351)	1.48 ± 0.51	1.46 ± 0.58	0.733
Zva, mm Hg/mL/m^2^ (n = 345)	5.64 ± 1.66	5.94 ± 2.0	0.152
**LV systolic function**			
Stroke volume, mL	57 ± 17	54 ± 18	0.111
Stroke volume index, mL/m^2^	32 ± 8	29 ± 8	0.002
Cardiac output, L/min	4.1 ± 1.0	4.0 ± 1.1	0.367
Cardiac Index, L/min/m^2^	2.3 ± 0.5	2.2 ± 0.5	0.011
Heart rate, bpm	73 ± 12	76 ± 14	0.059
LV systolic pressure, mm Hg (n = 366)	172 ± 29	163 ± 31	0.010
LV end-diastolic pressure, mm Hg (n = 366)	15 ± 7	18 ± 8	0.001
**Right heart hemodynamics**			
Pulmonary arterial wedge pressure, mm Hg	12 ± 6	17 ± 8	< 0.001
Pulmonary arterial systolic pressure, mm Hg	38 ± 12	49 ± 16	< 0.001
Pulmonary arterial diastolic pressure, mm Hg	12 ± 6	17 ± 8	< 0.001
Pulmonary arterial mean pressure, mm Hg	22 ± 8	29 ± 10	< 0.001
Pulmonary arterial compliance, mL/mm Hg	2.4 ± 1.0	2.0 ± 1.2	0.005
Transpulmonary pressure gradient, mm Hg	9 ± 5	12 ± 6	< 0.001
Pulmonary vascular resistance, mm Hg·min/mL	2.6 ± 1.8	3.4 ± 2.2	< 0.001

AUC, area under the curve; AVA, aortic valve area; bpm, beats per minute; BSA, body surface area; LV, left ventricle; mPA, main pulmonary artery; RV, right ventricle; RWT, relative wall thickness; TAPSE, tricuspid annular plane systolic excursion; TDI, pulsed-wave Doppler peak velocity at the tricuspid annulus; Zva, valvulo-arterial Impedance.

Aortic systolic blood pressure, pulse pressure, and mean blood pressure were significantly lower in the mPA_e_ group, whereas total arterial compliance and total vascular resistance did not differ significantly (Table 3). Stroke volume index (*P* = 0.002) and cardiac index (*P* = 0.011) were lower in the mPA_e_ group. Patients in the mPA_e_ group had an impaired pulmonary hemodynamic profile, with significantly higher systolic, diastolic, and mean PAP, elevated PAWP, and increased pulmonary vascular resistance than those in the mPA_n_ group (all *P* < 0.001; Table 3).

### TAVR intervention

The characteristics of the TAVR procedure are presented in Table 4. Femoral access was used in most patients (95.5%). A total of 41 patients (12.1%) underwent a concomitant procedure (coronary angioplasty). Device success was achieved in 316 interventions (93.7%), with comparable rates between the groups (*P* = 0.821).

**Table 4:** Transcatheter aortic valve replacement procedural characteristics

Characteristics	Normal mPA	Dilated mPA	*P*
	*n = 145*	*n = 191*	
**Access site**			0.729
Femoral	139 (95.9)	183 (95)	
Apical	2 (1.4)	3 (2)	
Sub-clavian	2 (1.4)	5 (2)	
Other	2 (1.4)	1 (1)	
**Prosthetic valve type**			0.006
Medtronic CoreValve (Medtronic, Mineapolis, MN)	123 (85)	179 (93)	
Edwards Sapien (Edwards Lifesciences, Irvine, CA)	17 (12)	13 (7)	
Boston Acurate (Boston Scientific, Marlborough, MA)	5 (3)	0 (0)	
**Procedural specifications**			
Concomitant procedure	15 (10)	26 (14)	0.405
Device success	135 (93)	181 (94)	0.821

Values are n (%), unless otherwise indicated.

mPA, main pulmonary artery.

### Invasive PA pressure vs mPA diameters

All CTA-derived geometric characteristics of the PA demonstrated significant correlations with the mean PAP, with the mPA diameter exhibiting the highest correlation coefficients (Pearson *r* = 0.393, *P* < 0.001; Table 5). The mPA diameter also was associated significantly with systolic (Pearson *r* = 0.379, *P* < 0.001) and diastolic PAP (Pearson *r* = 0.373, *P* < 0.001), as well as PAWP (Pearson *r* = 0.315, *P* < 0.001). mPA diameter also was associated with pulmonary vascular resistance (Pearson *r* = 0.202, *P* < 0.001). Interestingly, mPA diameter was inversely correlated with right ventricular longitudinal performance indexes (TDI, Pearson *r* = –0.151, *P* = 0.006; tricuspid annular plane systolic excursion, Pearson *r* = –0.118, *P* = 0.032) and positively associated with left atrial volumes (Pearson *r* = 0.414, *P* < 0.001).

**Table 5:** Correlations between invasively measured mean pulmonary artery pressure (PAP) and radiographic main pulmonary artery (mPA) measures

Measure	Mean PAP	*P*
	Pearson *r*	
Axial mPA diameter	0.393	< 0.001
Main PA diameter A (MPR)	0.365	< 0.001
Main PA diameter B (MPR)	0.392	< 0.001
Main PA periphery (MPR)	0.385	< 0.001
Main PA surface area (MPR)	0.376	< 0.001

MPR, multiplanar reformation; PA, pulmonary artery.

### Clinical outcomes

Clinical follow-up was completed for the entire study population. Mortality data stratified by mPA diameter are presented in the Central Illustration (Kaplan–Meier and Cox regression analyses). Compared to patients with a normal mPA, those with an enlarged mPA exhibited a higher overall mortality rate at 1 year (Kaplan–Meier log rank *P* = 0.022, unadjusted HR 2.59; 95% CI: 1.11-6.04, *P* = 0.027), which remained significant even after adjustment for baseline characteristics (adjusted HR 2.82; 95% CI: 1.18-6.77, *P* = 0.02). When mPA values were normalized to the aortic diameter, the associations with 1-year mortality became stronger (Kaplan–Meier log rank *P* < 0.001, unadjusted HR 3.83; 95% CI: 1.85-7.87, *P* < 0.001, adjusted HR 4.2; 95% CI: 2.0-8.98, *P* < 0.001). Patients with invasively-defined PH exhibited higher 1-year all-cause mortality rates than those without invasively-defined PH, as expected (unadjusted HR 2.83; 95% CI: 1.39-5.76, *P* = 0.004). Key findings are summarized in the Central Illustration.

Additional analyses using mPA diameter indexed to BSA showed a significant positive correlation with mean PAP (Pearson *r* = 0.391, *P* < 0.001). For prognostic assessment, and in the absence of established cutoff values, patients in the upper tertile of PA/BSA were compared with the remainder of the cohort. This group demonstrated a significantly higher mortality risk (Cox regression HR 2.1, 95% CI: 1.02-4.28, *P* = 0.043).

## Discussion

The main findings of the present study may be summarized as follows: (i) In patients with severe AS undergoing TAVR, PA enlargement (mPA diameter > 29 mm for male patients and > 27 mm for female patients), as assessed by CTA, was independently associated with higher 1-year all-cause mortality compared to normal mPA size. This association remained significant even after adjustment for baseline characteristics and became stronger when mPA diameter was indexed to the ascending aorta. (ii) The prognostic significance of CTA-derived mPA enlargement was comparable to the PH status defined by invasive mean PAP. (iii) CTA-derived mPA diameter was strongly associated with RV longitudinal function assessed by TDI and left atrial volumes, reflecting the complex pathophysiology of PH in severe AS.

PH is highly prevalent in the TAVR population, affecting up to three-quarters of patients referred for TAVR, and is consistently associated with excess mortality, particularly for precapillary and combined patterns.^1^^,^^2^ In our cohort, the 1-year mortality incidence among patients with PH was comparable to that reported in previous studies.^1^^,^^17^ To date, most studies have assessed PH using RHC or transthoracic echocardiography, whereas only a few have evaluated PA enlargement, an anatomic consequence of PH.

An important finding in our work is that mPA enlargement proved to be a powerful prognostic marker, with risk persisting after adjustment for baseline comorbidities. Our findings are consistent with previous reports. Koseki et al.^13^ showed that CTA-derived mPA dilatation (≥ 29 mm) was independently associated with 2-year mortality in nearly 900 TAVR patients (adjusted HR 2.21), particularly in those with the highest pulmonary pressures. Similarly, Turner et al. demonstrated that mPA area on CTA predicted 1-year mortality (HR 2.04 per standard deviation), with significantly lower survival above a threshold of 7.4 cm^2^.^12^ Collectively, these results emphasize the prognostic relevance of mPA enlargement across various measurement strategies.

Our study further highlights the incremental prognostic value of indexing mPA diameter to the ascending aorta. Although mPA enlargement alone conferred a 2.6-fold increased mortality risk, an mPA/Ao ratio > 0.91 was associated with a nearly 4-fold risk (HR 3.83). This observation aligns with the report by Hakgor et al.,^18^ who demonstrated that an mPA/aorta (Ao) ratio > 0.86 independently predicted both in-hospital and 2-year mortality, with an HR of 3.697 (95% CI: 2.341-5.840; *P* < 0.001). Thus, normalization of the mPA size to the ascending aortic diameter provides a more discriminant prognostic marker and identifies a particularly high-risk subset. These convergent results underscore the clinical utility of this simple and reproducible index for refining risk stratification in TAVR candidates. Nevertheless, the optimal threshold is still debated. In our study, we used a cutoff of 0.91, with findings consistent with those of Hakgor et al., although their cutoff was 0.86.^18^ Taken together, these data suggest that although cutoffs may vary slightly across cohorts, the ratio consistently identifies high-risk patients. Validation in larger prospective series is warranted.

Indexing mPA size to BSA may represent an approach to account for interindividual variability unrelated to PH. Indeed, prior data (eg, from the Framingham cohort) have shown a moderate correlation between mPA size and BSA.^11^ Current imaging recommendations, including those from the Fleischner Society, do not advocate routine normalization of mPA diameter to BSA in the assessment of PH, and absolute or mPA-to-aorta ratio measurements remain the most used parameters.^19^ In our cohort, these additional analyses showed a significant positive correlation with mean PAP. The upper tertile of mPA/BSA was associated with a significantly higher risk of mortality.

The pathophysiological mechanisms underlying PA enlargement are multifactorial. Chronic exposure to elevated pulmonary pressures drives vascular remodelling, stiffening, and PA dilatation. This process is typically accompanied by right ventricular dysfunction, tricuspid regurgitation, and reduced pulmonary compliance, all of which independently worsen outcomes after TAVR.^20^^,^^21^ Although RHC provides an accurate assessment of PAP, it represents an instantaneous measure that depends on loading conditions or rhythm disturbances. In contrast, mPA dilatation reflects the cumulative effect of chronically elevated pressures. As the mPA enlarges only in response to sustained overload, it provides a more stable and integrated marker of long-term pulmonary pressures, which explains its strong prognostic value. However, although mPA dilatation reflects the presence of PH, it does not indicate its underlying phenotype (pre, postcapillary, or combined). Notably, distinct hemodynamic patterns, particularly precapillary and combined, are associated with a worse prognosis.^1^ Finally, in our cohort, mPA enlargement clustered with comorbidities such as atrial fibrillation, COPD, and diabetes, which likely accelerate pulmonary vascular remodelling and further contribute to excess mortality. Nevertheless, mPA enlargement remained significant after adjustment, highlighting its potential as a surrogate marker for prognosis.

Although RHC remains the gold standard for PH diagnosis, it is invasive and time-consuming, and therefore is not routinely performed before TAVR. Echocardiography is routinely used for noninvasive screening. Although the use of echocardiography-derived right ventricular systolic pressure to estimate PAP is clinically appealing, several limitations have been reported. Its accuracy may be hampered by acoustic windows, and the right ventricular systolic pressure estimation may be significantly influenced by right ventricular function and the presence of severe tricuspid regurgitation, leading to potential over- or underestimation. In contrast, CTA is systematically performed in all TAVR candidates for annular sizing and vascular access planning, and mPA measurement requires no additional imaging. Furthermore, echocardiography is more operator-dependent and probably less reproducible than CT scan measurement. In our study, all patients underwent RHC before TAVR, unlike previous cohorts for which invasive assessment was absent or selective. This comprehensive evaluation not only validates the strong correlation between CTA-derived mPA size and invasive hemodynamics but also strengthens the robustness of our conclusions, reinforcing the clinical relevance of this marker. Our results, therefore, support the routine use of mPA enlargement, particularly the mPA/Ao ratio, as a readily available surrogate for PH in this context.

From a clinical perspective, these findings carry important implications. Current risk models, such as the Society of Thoracic Surgeons score, do not incorporate pulmonary vascular parameters, despite the well-established prognostic role of PH in TAVR. Incorporating mPA enlargement into pre-procedural assessment, especially when indexed to the ascending aorta, may refine risk stratification and expected benefit from TAVR and could help identify patients who require closer follow-up, optimization of comorbidities, or tailored peri-procedural management.

### Limitations

Several limitations should be acknowledged. First, as this was a retrospective, single-centre study, residual confounding cannot be excluded entirely despite multivariable adjustment. However, CT scan measurements of mPA and Ao diameters were performed by trained radiologists who were blinded to the RHC results. Second, mPA enlargement is not specific to PH and may be influenced by concomitant pulmonary or systemic diseases. Still, its strong and independent association with mortality supports its value in real-world practice. Third, we did not assess post-TAVR changes in mPA size, which may provide further prognostic insights. Fourth, echocardiography-derived estimates of right ventricular systolic pressure were available only in a limited subset of the study population, thereby limiting their use for subgroup comparisons and correlations with mPA dimensions. Fifth, given that AS worsening and mPA dilatation are slow processes, the potential impact of the median 21-day interval between RHC and CTA on the observed correlations could not be assessed specifically. Finally, although our systematic use of RHC adds strength, external validation in larger multicentre cohorts is warranted before incorporating mPA measurements into routine clinical decision-making.

## Conclusion

Pre-procedural CTA-derived mPA enlargement, particularly when indexed to the ascending aortic diameter, is strongly associated with 1-year all-cause mortality after TAVR. These results suggest that mPA size, a simple and noninvasive imaging marker, could be integrated into risk-stratification algorithms to better identify high-risk patients with severe AS undergoing TAVR.

## Ethics Statement

The research reported in this paper adhered to the CONSORT guidelines.

## Patient Consent

The authors confirm that a patient consent form has been obtained for this article.

## Funding Sources

The fees for publication are supported by the 10.13039/501100004944Naresuan University.

## Disclosures

S.N. reports being a consultant for Medtronic and receiving an institutional grant from Edwards LifeSciences. The other authors have no conflicts of interest to disclose.
